# Evaluating the Effectiveness of Fish Consumption Advisories: Modeling Prenatal, Postnatal, and Childhood Exposures to Persistent Organic Pollutants

**DOI:** 10.1289/ehp.1206380

**Published:** 2013-12-17

**Authors:** Matthew J. Binnington, Cristina L. Quinn, Michael S. McLachlan, Frank Wania

**Affiliations:** 1 Department of Physical and Environmental Sciences, University of Toronto Scarborough, Toronto, Ontario, Canada; 2 Department of Applied Environmental Science (ITM), Stockholm University, Stockholm, Sweden

## Abstract

Background: Because human exposure to persistent organic pollutants (POPs) occurs mainly through ingestion of contaminated food, regulatory bodies issue dietary consumption advisories to describe safe intake levels for food items of concern, particularly fish.

Objectives: Our study goal was to estimate the effectiveness of fish consumption advisories in reducing exposure of infants and children to POPs.

Methods: We used the time-variant mechanistic model CoZMoMAN to estimate and compare prenatal, postnatal, and childhood exposure to polychlorinated biphenyl congener PCB-153 under different scenarios of maternal guideline adherence for both hypothetical constant and realistic time-variant chemical emissions. The scenarios differed in terms of length of compliance (1 vs. 5 years), extent of fish substitution (all vs. half), and replacement diet (uncontaminated produce vs. beef). We also estimated potential exposure reductions for a range of theoretical chemicals to explore how guideline effectiveness varies with a chemical’s partitioning and degradation properties.

Results: When assuming realistic time periods of advisory compliance, our findings suggest that temporarily eliminating or reducing maternal fish consumption is largely ineffective in reducing pre- and postnatal exposure to substances with long elimination half-lives in humans, especially during periods of decreasing environmental emissions. Substituting fish with beef may actually result in higher exposure to certain groups of environmental contaminants. On the other hand, advisories may be highly effective in reducing exposure to substances with elimination half-lives in humans shorter than the length of compliance.

Conclusions: Our model estimates suggest that fish consumption advisories are unlikely to be effective in reducing prenatal, postnatal, and childhood exposures to compounds with long elimination half-lives in humans.

Citation: Binnington MJ, Quinn CL, McLachlan MS, Wania F. 2014. Evaluating the effectiveness of fish consumption advisories: modeling prenatal, postnatal, and childhood exposures to persistent organic pollutants. Environ Health Perspect 122:178–186; http://dx.doi.org/10.1289/ehp.1206380

## Introduction

For most individuals worldwide, exposure to most persistent organic pollutants occurs mainly through ingestion of contaminated food (Moser and McLachlan 2002; Patandin et al. 1999). In response, governmental agencies issue dietary consumption advisories to suggest safe intake levels of specific items for their citizens (e.g., Health Canada 2009; Ontario Ministry of the Environment 2011). A particular focus is put on fish consumption, because fish are a major route of exposure to bioaccumulating contaminants (Turyk et al. 2012). Although most fish consumption advisories are developed to reduce methylmercury exposure (Cohen et al. 2005; Health Canada 2009), several are provided explicitly for persistent organic pollutants (POPs). For example, Canadian advisories designed for the Great Lakes sport fishing community are based on contamination by polychlorinated biphenyls (PCBs), which are of greater concern to this population than contamination from mercury (Bhavsar et al. 2011). Also, the European Union (EU) requires Finnish and Swedish governmental agencies to provide dietary restrictions for Baltic herring because of levels of polychlorinated dibenzo-*p*-dioxins and dibenzofurans (PCDD/Fs) and PCBs that might result in exposures above maximum total weekly consumption guidelines (European Commission 2006; Kiljunen et al. 2007). Susceptible populations are recommended to limit intake of Baltic herring to 2–3 times per year (Sweden National Food Administration 2008).

Concern about human exposure to PCBs is attributable in large part to their neurocognitive toxicity; effects that have been linked to non-dioxin-like PCB exposure during early life include reduced IQ, growth impairment, and decreased motor skills (Jacobson et al. 1990; Stewart et al. 2008; Walkowiak et al. 2001). Also, reproductive deficits such as abnormal development and decreased fecundity later in life have been associated with non-dioxin-like PCB exposures (Guo et al. 2000; Hsu et al. 2003; Toft et al. 2004, 2006). Thus, many consumption advisories based on PCBs and other chemicals of neurodevelopmental concern such as methylmercury focus on women of childbearing age—particularly pregnant or nursing mothers and young children. PCB-based advisories typically promote reducing intake of large lipid-rich fish, such as freshwater salmon, trout, carp, and catfish; members of sensitive populations are advised to trim fatty areas before consumption as well as to avoid fish roe, due to high partitioning of PCBs into these tissues. (Ontario Ministry of the Environment 2011). Specific advisories for methylmercury suggest limiting consumption of large predatory fish [U.S. Food and Drug Administration (FDA) 2004]. For example, Health Canada (2009) guidelines recommend a piscivorous fish consumption limit of 150 g/month for pregnant or nursing mothers.

Because of the toxicity of dioxin-like congeners, PCBs are also classified as probable human carcinogens (Group 2A) by the International Agency for Research on Cancer (IARC 2009), with PCB-126 (3,3´,4,4´,5-pentachlorobiphenyl) further listed as carcinogenic to humans (Group 1) (IARC 2012). For this reason, additional advisories are published for fish consumers outside populations sensitive to neurodevelopmental PCB effects, such as members of the greater sport fishing community. Several studies have attempted to quantify associated cancer risks among fish consumers (Brustad et al. 2007; McElroy et al. 2004), but have yet to detail a statistically significant association between sport fish consumption and cancer end points. In fact, Stone and Hope (2010) advocated against providing fish advisories based exclusively on POP-related cancer concerns mainly because of the health benefits of fish intake as well as the excessively conservative estimates of cancer risk compared with these potential health gains. Specifically, fish are an excellent source of omega-3 polyunsaturated fatty acids, which are essential for healthy neurological development and maintaining cardiovascular fitness (Kris-Etherton et al. 2002, 2007; Lee et al. 2009) as well as several nutrients, particularly selenium, vitamin D, and vitamin E (Becker et al. 2007).

Several published evaluations of fish consumption advisory efficacy have assessed individuals’ awareness of recommendations (Burger and Gochfeld 2008, 2009), evaluated individual compliance by measuring behavioral changes (Teisl et al. 2011), and also whether the competing benefits of fish consumption are sufficiently considered by regulators (Scherer et al. 2008). However, little attention has been given to the actual declines in human contaminant exposure expected from following present advisories. Thus, our objective in the present study was to quantitatively estimate the efficacy of current fish consumption advisories for mothers in reducing PCB exposure of their children during identified life stages of concern: the prenatal, postnatal, and childhood periods. A second goal was to explore how chemical partitioning and degradation properties might influence exposure reductions among women following advisories. To pursue these objectives, we employed a mechanistic model to estimate exposures in individuals based on predicted PCB concentrations in environmental media and subsequent bioaccumulation through terrestrial and aquatic food chains. Principally, we examined the potential influence of the length and degree of advisory compliance, the pattern of environmental emissions, and the partitioning properties and human metabolic transformation half-life of chemicals on the effectiveness of consumption guidelines in reducing exposures.

## Methods

*Modeling approach and defining of default parameters*. We performed simulations using the time-variant contaminant fate model CoZMoMAN (Breivik et al. 2010) to estimate prenatal, postnatal, and childhood exposures to the non-dioxin-like congener PCB-153 (2,2´,4,4´,5,5´-hexachlorobiphenyl) and to a suite of hypothetical chemicals among women differentiated by their compliance with selected fish consumption advisories. CoZMoMAN combines the environmental multimedia transport and distribution model CoZMoPOP2 (Wania et al. 2006) with the human food chain bioaccumulation model ACC-Human (Czub and McLachlan 2004b) and is presently parameterized for southern Sweden. Representative for a typical southern Swedish population, major dietary exposure vectors of organic contaminants included in the model are herring, cod, beef, and dairy products. CoZMoMAN was initially evaluated by reproducing measured time-variant concentrations of PCBs in environmental compartments, local food items, and human breast milk in southern Sweden (Breivik et al. 2010; Czub and McLachlan 2004b). For PCB-153 specifically, estimates in all environmental and biotic compartments were within a factor of 2 of measured values (Breivik et al. 2010).

For the present analysis, we modeled exposures for a hypothetical woman born to a 30-year-old mother who then gave birth to her own child at 30 years of age. We calculated female growth curves according to Moser and McLachlan (2002) and estimated prenatal and postnatal contaminant transfers by assuming that infants and breast milk were at chemical equilibrium (at equifugacity) with the body tissue and blood of the mother at the time of birth and during lactation, respectively. Further, we assumed that infants nursed for 6 months, after which they ate a regular diet corrected for age (Moser and McLachlan 2002; Quinn et al. 2011). We estimated food consumption rates from dietary statistics for 25-year-old Swedish women between 1930 and 2006. Consumption was highest in 2006 (912 g animal products/day) and was assumed to be constant thereafter. This maximum intake was composed of 792 g dairy/day, 44 g beef/day, and 76 g fish/day (57 g herring/day, and 19 g cod/day). At assumed lipid fractions for each food item (dairy, 5.5%; beef, 26.0%; herring, 3.5%; cod, 0.5%), the estimated maximum daily lipid intake was 57 g lipid/day. We then calculated age-scaled food consumption rates based on the estimated rates for 25-year-old women. We assumed that the sources of the consumed lipids for the 25-year-old female remained constant through time at 76% dairy lipid, 20% beef lipid, and 4% fish lipid, and we also assumed that these proportions did not change with age. We described human metabolic transformation of the contaminants as a linear first-order elimination process with a rate constant that remained unchanged throughout life (i.e., 5.25 × 10^–6^/hr) for PCB-153, corresponding to a 15.1-year half-life) (Czub and McLachlan 2004b). We used the model output of lipid-normalized human contaminant concentration at birth (nanograms per gram lipid) to assess prenatal exposure, and postnatal and childhood exposures were defined as the average concentrations encountered from 0–6 months (the duration of breastfeeding) and 0–9 years, respectively. Because the latter two were calculated as the area under the concentration–age curve, they have units of nanograms per gram lipid·year.

*Defining scenarios of fish consumption guideline compliance*. As discussed, fish consumption advisories often target populations designated as sensitive to the deleterious neurological effects of PCB exposure, particularly those within major developmental stages: prenatal, postnatal, and childhood growth. Because specific advisories vary quantitatively in recommended daily fish intake based on the target community, we adopted the framework outlined in the *Guide to Eating Ontario Sport Fish* based on its simplicity; generally, children and women of childbearing age are advised to regularly consume half or none of the suggested serving sizes for the general population, which vary among fish of different species, size, and lake (Ontario Ministry of the Environment 2011). Per these recommendations, we compared model results for females consuming a typical Swedish fish diet [age- and time-scaled based on daily consumption by a 25-year-old female in 2006 of 57 g herring/day (75%) and 19 g cod/day (25%)] (Czub and McLachlan 2004b), to those replacing half the default serving of fish (38 g/day) or replacing all fish (76 g/day). Although fish consumption advisories recommend adherence throughout the full extent of childhood and childbearing age, we expect most individuals to merely comply with these guidelines for a variable period before and during pregnancy and breastfeeding, reflecting the short-term guideline compliance behavior documented by Teisl et al. (2011). For this reason, we assumed that modeled females followed fish consumption advisories only for 1.5 years—the 3 months before pregnancy, plus the 9-month gestation and 6-month breastfeeding periods. We also performed calculations that assumed compliance periods of 5 years before birth as well as the entire 30-year period before birth, to estimate the impact of long-term and maximal compliance periods, respectively.

We assumed that women reducing fish intake replaced this dietary portion by one of two distinct scenarios: with either an increase in noncontaminated food (termed “produce”) because fruits and vegetables are not a significant vector for human PCB exposure (De Mul et al. 2008; Zohair et al. 2006), or a proportional increase in beef consumption—direct replacement of one modeled meat product with another. Beef contaminant concentrations were calculated mechanistically within CoZMoMAN by quantifying the transfer from air to grass to cow. Daily fish consumption was reduced on a gram wet weight basis for the produce-substitution scenarios, and fish was replaced on a gram-for-gram (wet weight) basis in the beef-substitution diets. This resulted in contaminated food intake by mothers under each replacement scenario that was proportional to the following maximal levels of consumption for 25-year-olds: replacing half of the fish with produce, at 874 g animal products/day (91% dairy, 5% beef, 4% fish); replacing half of the fish with beef, at 912 g animal products/day (87% dairy, 9% beef, 4% fish); replacing all fish with produce, at 836 g animal products/day (95% dairy, 5% beef); replacing all fish with beef, at 912 g animal products/day (87% dairy, 13% beef).

*Calculations for PCB-153 assuming hypothetical constant or historical time-variant emissions*. We focused primarily on PCB-153 because previous CoZMoMAN estimates of this congener exhibited the greatest consistency with measured time trends in the Western Baltic drainage basin (Breivik et al. 2010). Plus, this congener’s extreme recalcitrance in humans likely represents a worst-case scenario for the potential of temporary dietary changes to reduce resultant human exposure. Our initial calculations for PCB-153 assumed constant emissions through time, resulting in steady-state concentrations in the environment, dietary items, and ultimately humans as well, such that generational differences in exposure (i.e., differences between mothers and their children) would be ≤ 1%. This simplifying assumption allowed us to estimate the effectiveness of fish consumption advisories in the absence of temporal changes in environmental PCB-153 contamination.

Our next set of simulations accounted for variation in PCB-153 emissions over time, which were estimated based on a well-evaluated historic emissions inventory for southern Sweden (Breivik et al. 2010). Specifically, PCB-153 emissions increased from the 1940s–1970s, peaked during 1975, and subsequently declined following Swedish restrictions on PCB manufacturing and use legislated during the 1970s (Breivik et al. 2002a, 2002b). To demonstrate the influence of increasing and decreasing contaminant emissions on the efficacy of consumption guidelines, we estimated PCB-153 exposures for women born every 5 years from 1950 through 2010, applying the same default model parameters and guideline adherence scenarios used for calculations assuming steady-state emissions.

*Steady-state emissions scenario for hypothetical chemicals*. We further explored the potential exposure reductions associated with fish consumption guidelines for a range of hypothetical contaminants that differed by their chemical partitioning and degradation properties. Calculations of this nature allow identification of the group(s) of contaminants for which adjustments of fish intake elicit the greatest effect on human exposure. We performed simulations for multiple combinations of octanol–air (*K*_oa_) and octanol–water (*K*_ow_) partition coefficients that define the chemical partitioning space. According to Czub and McLachlan (2004a), the area of the chemical space containing contaminants with an elevated bioaccumulation potential is roughly bordered by 2 < log *K*_ow_ < 11 and log *K*_ow_ > 7. Therefore, we focused on this area of the chemical partitioning space in our calculations; log *K*_ow_ was varied from 2 to 12 and log *K*_oa_ from 7 to 12 by half log-unit increments. From an analysis of dietary bioaccumulation potential by Undeman et al. (2010), we expected exposure to contaminants with a log *K*_ow_ of 6 to 9 and a log *K*_oa_ of 9 to 12 [e.g., dichlorodiphenyltrichloroethane (DDT), as well as several highly chlorinated PCBs, PCDDs, PCDFs] to be most affected by advisory compliance because fish intake was estimated to result in > 50% of the exposure to chemicals possessing these properties. For our chemical partitioning space analyses, we assumed that compounds were emitted at a constant rate directly to the atmosphere, and we then calculated exposure reductions once human concentrations reached steady state. Initial simulations assumed that chemicals were perfectly persistent, exhibiting negligible breakdown in environmental compartments and organisms (including humans), which allowed us to evaluate the influence of chemical partitioning properties and compliance scenarios in the absence of any modifying effects of metabolism. In other words, we simulated constant chemical emissions until no difference in contaminant exposure was estimated between human generations (steady-state assumption), and contaminants were also not subject to any degradation processes in environmental and biotic compartments (perfect persistence assumption).

Next, we extended our chemical partitioning space simulations by allowing the rate constant for metabolic breakdown in humans to vary, using values corresponding to biotransformation half-lives of 1, 3, 5, and 15 years. Steady-state emissions were maintained, and we continued to assume perfect chemical persistence in all model compartments and organisms other than humans. We also used the exposure changes calculated for hypothetical chemicals to estimate those for real advisory-relevant POPs by placing them on graphs of the results according to their measured partitioning properties and human elimination half-lives (Åberg et al. 2008; Geyer et al. 2002, 2004; Li et al. 2003; Milbrath et al. 2009; Ritter et al. 2009, 2011; Schenker et al. 2005). We also conducted *post hoc* analyses to compare fish advisory effectiveness assuming an organic chemical biotransformation half-life approaching that of the elimination half-life for methylmercury (40–50 days) (Stern 2005), because methylmercury represents the target compound for many dietary fish advisories. All chemical partitioning space calculations were limited to an evaluation of the effect of replacing all fish consumption with produce for 5 years before childbirth on prenatal chemical exposure.

## Results

**Figure 1:**
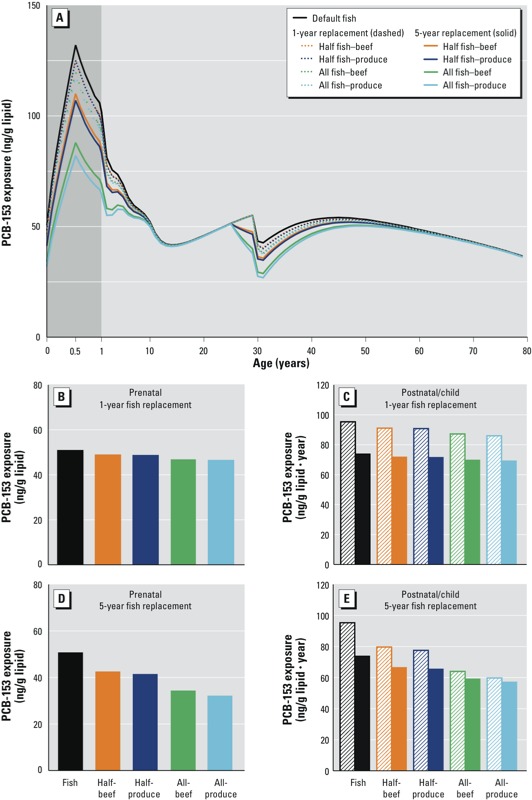
Estimated absolute reductions in prenatal, postnatal, and childhood exposure to PCB-153 according to different maternal fish advisory compliance scenarios (relative to no change in fish consumption), and assuming steady-state emissions. Concentration age profiles for each compliance scenario are depicted for 1- and 5-year adherence (*A*). Exposure profiles are the same for all generations at steady state, and thus the lifetime trends of PCB-153 contamination for a mother and her child under each scenario are depicted on the same graph. Prenatal peak exposure estimated for 1- and 5-year scenarios are depicted as solid bars in (*B*) and (*D*), respectively. Postnatal and childhood exposures under 1- and 5-year compliance are displayed in (*C*) and (*E*), respectively; the hatched bars represent postnatal exposures, and the solid bars represent childhood exposures. Also, prenatal exposures in (*B*) and (*D*) are point estimates of PCB-153 body burden at birth, whereas postnatal and childhood exposures in (*C*) and (*E*) are the time-integrated areas under the curve during individuals’ first 6 months and first 9 years of life, respectively.

*Fish advisory impact assuming steady-state emissions of PCBs*. Figure 1A compares the PCB-153 concentration–age relationships for each of the five fish consumption scenarios assuming compliance for either a 1-year or 5-year period before childbirth. As described in detail by Quinn et al. (2011), under the steady-state emissions assumption, exposures to mothers and their children can be represented using a single age continuum because there are no differences between generations in exposure at any given age. Therefore, at older ages, differences in PCB-153 concentrations relative to those estimated for default fish consumption represent the impact of changes in a mother’s fish consumption on her own exposure, whereas exposures at younger ages represent the estimated impact of changes in a mother’s fish consumption on her child’s exposure (Figure 1A). Model estimates suggest that following consumption guidelines for 1 year before childbirth would result in negligible reductions in prenatal PCB-153 exposure (represented by lipid-normalized concentration estimates at birth; Figure 1B), and in postnatal and childhood exposure (represented by annual average concentrations during the 6-month nursing period, and from birth through 9 years of age; Figure 1C) relative to exposures estimated assuming no changes in maternal fish consumption. More specifically, for all 1-year fish replacement scenarios, decreases in PCB-153 concentrations were < 10% across the three relevant exposure periods. The greatest declines in exposure following fish replacement for 1 year were obtained for the scenario total fish replacement with produce [4 ng/g lipid (9%), 9 ng/g lipid·year (10%), and 5 ng/g lipid·year (6%) for prenatal, postnatal, and childhood exposures, respectively], with slightly smaller declines following total fish replacement with beef (Figure 1B,C). Declines were greater following 5 years of maternal compliance with fish consumption guidelines, yet only scenarios assuming total fish replacement generated appreciable exposure reductions (≥ 30%) [e.g., for total replacement with produce: 19 ng/g lipid (37%), 36 ng/g lipid·year (37%), and 17 ng/g lipid·year (23%) for prenatal, postnatal, and childhood exposures, respectively]; slightly smaller declines were again estimated for total fish replacement with beef (Figure 1D,E).

We also performed estimates for a best-case scenario of lifetime guideline adherence because dietary POP exposure during childhood has been shown to contribute significantly to body burdens among children (Burns et al. 2009). When fish consumption was reduced throughout the entire 30-year period before childbirth, total fish replacement with produce resulted in PCB-153 exposure declines of 43 ng/g lipid (85%), 81 ng/g lipid·year (85%), and 63 ng/g lipid·year (85%) for prenatal, postnatal, and childhood exposures, respectively (see Supplemental Material, Figure S1). Smaller relative exposure declines of 75% were calculated for each of prenatal, postnatal, and childhood PCB-153 exposure assuming total lifetime fish replacement with beef. No difference among prenatal, postnatal, and childhood percent exposure declines was estimated for lifetime compliance because the diets of mothers and their children were assumed to be the same. Collectively, these exposure decline results for short-term, long-term, and lifetime fish consumption advisory compliance are consistent with the long human elimination half-life of PCB-153 (15.1 years). Additionally the 1-year compliance results suggest that contaminant load acquired before adhering to dietary adjustments would substantially offset the effect of decreased intake during short-term fish replacement.

**Figure 2:**
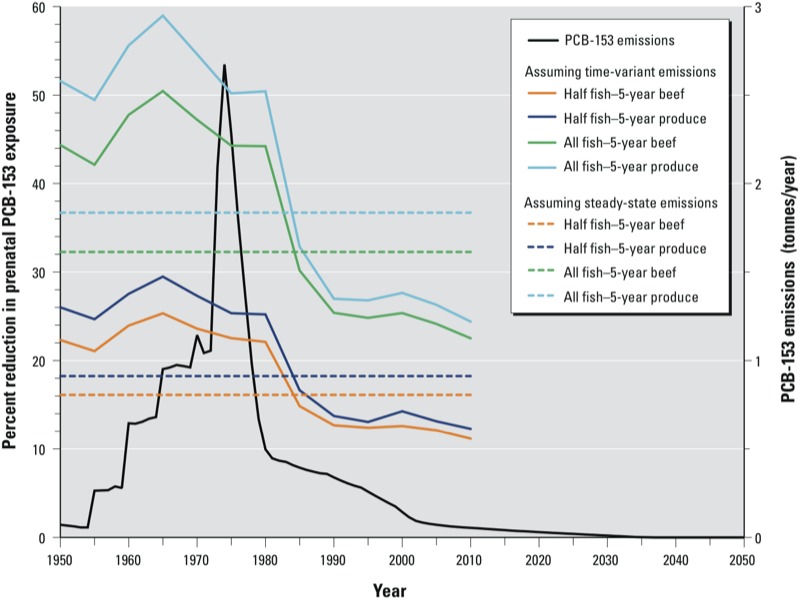
Estimated percent reductions in prenatal exposure to PCB-153 according to different maternal fish advisory compliance scenarios (relative to no change in fish consumption), and assuming time-variant emissions. The dotted lines represent the percent reduction in prenatal exposure for the same fish consumption scenarios described under steady-state conditions. Plots are overlaid atop the time-variant emissions scenario used in our simulations (Breivik et al. 2010). The same pattern of varying exposure reductions through time was also observed for postnatal PCB-153 exposures (data not shown).

*Fish advisory impact assuming time-variant emissions of PCBs*. To analyze the influence of time-variant emissions on PCB-153 exposures following maternal fish replacement, we focused on long-term (5-year) compliance scenarios because the 1-year compliance scenarios had limited impact when assuming steady-state emissions. Figure 2 shows, as a function of the child’s year of birth and the substitution scenario, the percent declines in prenatal exposure following maternal fish replacement during the 5 years before pregnancy assuming time-variant PCB-153 emissions. Variation in emissions over time markedly influenced the estimated impacts of fish replacement on exposures, with the greatest effects on prenatal exposures of children born during the time period of increasing emissions (7–57% for children born 1950–1970, depending on substitution scenario and compliance duration) and attenuated impacts on children born during the period of decreasing emissions (2–28% for children born 1990–2010). In fact, by the mid- to late 1980s, the estimated percent reductions in prenatal exposures for all 5-year replacement scenarios dropped below reductions estimated when steady-state emissions were assumed (Figure 2). This phenomenon was expected because children born to mothers replacing dietary fish intake during periods of increasing environmental contaminant concentrations would be avoiding a greater potential exposure than those born during periods of decreasing environment levels. For example, the estimated PCB-153 exposure of a child born in 1970 whose mother replaced all fish intake with produce for 5 years before birth was reduced relative to the default fish consumption scenario by 47 ng/g lipid (55%); the same scenario for a child born in 2000 resulted in an exposure reduction of 33 ng/g lipid (28%). This analysis illustrates that the time relative to peak emissions has a significant impact on the reduction in offspring exposure achieved by lowering maternal fish consumption.

Reductions in postnatal PCB-153 exposure are not displayed in Figure 2 because they closely mimic prenatal exposure reductions. Childhood PCB-153 exposure reductions are also not depicted because of a much less pronounced impact of maternal fish replacement scenarios on this end point, given the competing influence of childhood diets during the 8.5 years following weaning. For complete prenatal, postnatal, and childhood PCB-153 exposure reduction data according to child’s year of birth and substitution scenario, see Supplemental Material, Tables S1 (1-year replacement) and S2 (5-year replacement). Despite exhibiting a lesser impact of maternal fish replacement, childhood percent exposure declines also varied markedly with time when assuming time-variant PCB-153 emissions (Table S2). However, when temporal trends of childhood exposure reduction were compared with those for prenatal and postnatal exposure reductions, equivalent events (e.g., peak exposure declines) were delayed by the approximate duration of the childhood exposure period (9 years). Due to this 9-year effect delay, we calculated that the greatest impact of maternal fish replacement on childhood exposure reductions would actually occur during the period of decreasing environmental PCB-153 concentrations (1975–1980), unlike estimates of prenatal and postnatal exposure declines.

**Figure 3:**
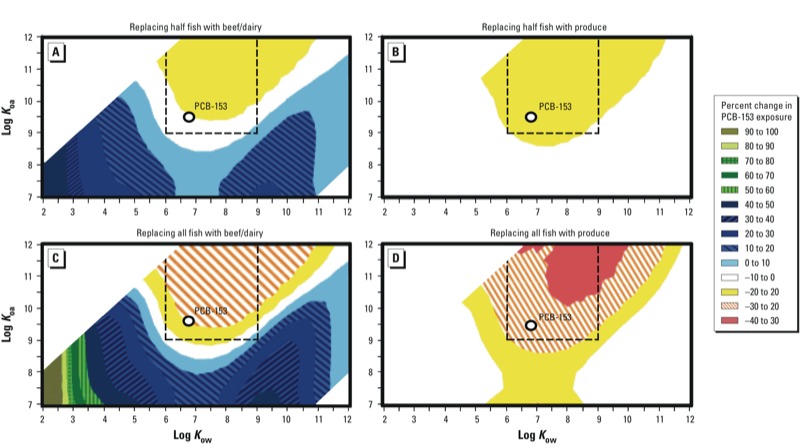
Estimated percent changes in prenatal exposure to perfectly persistent hypothetical chemicals with varying octanol–air (*K_oa_*) and octanol–water partition (*K_ow_*) coefficients at 25°C according to different maternal fish advisory compliance scenarios (relative to no change in fish consumption). Graphs represent the estimated percent change from the calculated default exposure following 5 years of (*A*) replacing half of fish intake with beef, (*B*) replacing half of fish intake with produce, (*C*) replacing all fish intake with beef, and (*D*) replacing all fish intake with produce, before pregnancy. Perfect persistence assumes no metabolic degradation of the chemical in any modeled organism (including humans); even though no real chemical satisfies this assumption, PCB-153 is shown in each of the plots as a reference. When a reduction in chemical exposure is estimated, the percent change is assigned a negative value (–40 to 0%); when an increase in exposure is estimated, the percent change is positive (0–60%). The area designated by the dashed line indicates the region of enhanced human bioaccumulation potential through the aquatic food chain identified by Undeman et al. (2010).

*The impact of fish consumption advisory compliance scenarios assuming steady-state emissions of hypothetical chemicals*. When contaminants were assumed to be perfectly persistent, model projections of prenatal exposure assuming 5-year advisory compliance and constant chemical emissions indicated both increasing and decreasing exposures, depending on chemical properties and the specific fish substitution scenario (Figure 3). For decreasing exposures, we observed the greatest impact of fish advisory compliance for chemicals within the region roughly bordered by a log *K*_ow_ of 5 to 11 and a log *K*_oa_ of 8.5 to 12, which includes POPs such as DDT, 2,3,7,8-TCDD (2,3,7,8-tetrachlorodibenzo-*p*-dioxin), PCB-126, and PCB-169. This region of chemicals also roughly corresponds to those with properties previously identified to possess elevated human bioaccumulation potential (Czub and McLachlan 2004a; Undeman et al. 2010). Specifically, prenatal exposures for these chemicals were estimated to decrease by 0–16% when replacing half of fish intake with beef or produce for 5 years (Figure 3A,B) and by 0–31% when replacing all dietary fish (Figure 3C,D). However, fish replacement with beef was also estimated to increase exposure to chemicals located in two regions: one bordered approximately by 2 < log *K*_ow_ < 6 and 7 < log *K*_oa_ < 9, the other by 8 < log *K*_ow_ < 10 and 7 < log *K*_oa_ < 9 (in blue, Figure 3A,C). Undeman et al. (2010) previously highlighted that cattle products are a more important vector of human exposure to persistent contaminants in these parts of the chemical partitioning space than the marine food chain. Moreover, Czub and McLachlan (2004a) identified the chemical uptake processes in vegetation that result in elevated cattle (and subsequently human) dietary bioaccumulation for both these chemical groups. For the chemical group bordered by 2 < log *K*_ow_ < 5 and 7 < log *K*_oa_ < 9, increased prenatal exposures are likely attributable to the strong biomagnification of persistent, hydrophilic, involatile chemicals in vegetation foliage following root uptake and translocation. For the second chemical group, bordered by 8 < log *K*_ow_ < 10 and 7 < log *K*_oa_ < 9, increased prenatal exposures likely result from the strong bioaccumulation of chemicals in vegetation through gaseous deposition.

**Figure 4:**
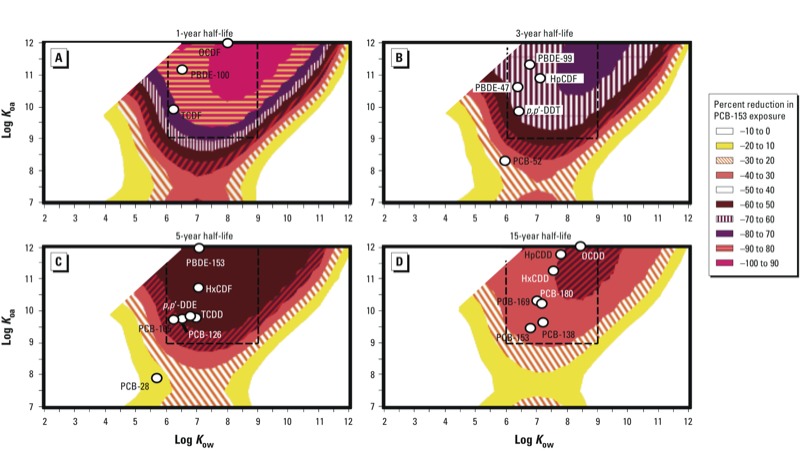
Estimated percent reductions in prenatal exposure to hypothetical chemicals with varying octanol–air (*K_oa_*) and octanol–water partition (*K_ow_*) coefficients at 25°C according to maternal fish advisory compliance, and assumed human biodegradation half-lives of (*A*) 1 year, (*B*) 3 years, (*C*) 5 years, and (*D*) 15 years. Graphs represent the estimated percent reduction from the calculated default exposure following 5 years of replacing all fish intake with produce before childbirth. Several POPs are placed in the plots according to their measured partitioning properties and human degradation half-lives. Abbreviations: DDE, dichlorodiphenyldichloroethylene; DDT, dichlorodiphenyltrichloroethane; HpCDD, 1,2,3,4,6,7,8-heptachlorodibenzo-p-dioxin; HpCDF1, 2,3,4,6,7,8-heptachloro-dibenzofuran; HxCDD, 1,2,3,4,7,8-hexachlorodibenzo-p-dioxin; HxCDF, 1,2,3,4,7,8-hexachlorodibenzofuran; OCDD, octachlorodibenzo-p-dioxin; OCDF, octachlorodibenzofuran; PBDE, polybrominated diphenyl ether; TCDD, 2,3,7,8-tetrachlorodibenzo-p-dioxin; TCDF, 2,3,7,8-tetrachlorodibenzofuran. (*D*) Also includes three contaminants with human biodegradation half-lives > 15 years (HxCDD, HpCDD, OCDD). Chemical partitioning properties were identified using data from Åberg et al. (2008), Li et al. (2003), and Schenker et al. (2005), and human degradation half-lives were compiled using data from Geyer et al. (2002, 2004), Milbrath et al. (2009), and Ritter et al. (2009, 2011). When a reduction in chemical exposure is observed, the percent change is assigned a negative value (–100 to 0%). The area designated by the dashed line indicates the region of enhanced human bioaccumulation potential through the aquatic food chain identified by Undeman et al. (2010).

When we modeled the effects of variation in chemical degradation as well as partitioning properties, we found that reductions in prenatal chemical exposure following dietary fish replacement increased as chemical metabolic half-lives decreased (Figure 4). Specifically, projected prenatal exposure declines for some chemicals within the region of enhanced human bioaccumulation potential through the aquatic food chain (log *K*_ow_ of 6 to 9 and log *K*_oa_ of 9 to 12, bordered by dashed lines in Figures 3 and 4) (Undeman et al. 2010) exceeded 90% following complete fish replacement with produce for 5 years when the human transformation half-life in humans was set to 1 year (Figure 4A). Comparative maximal prenatal exposure declines of 73%, 60%, and 42% were estimated when respective transformation half-lives of 3, 5, and 15 years were assumed (Figure 4B–D). Corresponding prenatal exposure declines for perfectly persistent chemicals following total fish substitution with produce are depicted in Figure 3D. Further, prenatal exposure reductions for organic chemicals with human biotransformation half-lives (90 days) approaching the elimination half-life of methylmercury (40–50 days) were predicted to closely mirror those for the 1-year transformation half-life estimates, with maximal declines reaching 96% (see Supplemental Material, Figure S2).

## Discussion

Throughout all our simulations, dietary fish substitution resulted in appreciable chemical exposure reductions to PCB-153, as well as chemicals expected to accumulate in humans based on their partitioning properties and biotransformation half-lives (e.g., 2,3,7,8-TCDD, PCB-126, PCB-169), only when long-term (5-year) advisory compliance was assumed. The lack of substantial impact for short-term compliance (1-year) suggests that current advisories based predominantly on long-lived POPs are likely to be ineffective in reducing human exposure to many chemical groups for which they are designed (i.e., PCBs, PCDDs). As an example, previous epidemiological work by Teisl et al. (2011) documented typical periods of maternal dietary change (limited to pregnancy and nursing) that were as brief as the short-term scenario we assumed. However, this study focused on dietary changes during pregnancy relative to prepregnancy, and during nursing relative to pregnancy. Even calculations assuming a period of 5-year compliance with fish advisories did not predict prenatal and postnatal exposure declines that fell below interindividual POP exposure differences observed in biomonitoring studies. For example, Schlummer et al. (1998) provide data for a small group of non-occupationally exposed individuals (*n* = 7), whose lipid-normalized PCB-153 blood lipid concentrations ranged over 7.1-fold (84–600 ng/g lipid). The greatest prenatal exposure reduction for PCB-153 we calculated was for children born in 1965 to mothers who completely replaced fish with produce for 5 years, assuming time-variant emissions. For these individuals we estimated a prenatal PCB-153 exposure decline of 59%, from 43 to 18 ng/g lipid, or merely a 2.4-fold decrease. Finally, our lifetime fish advisory compliance results suggest that periods of adherence that include childhood and childbearing age (Ontario Ministry of the Environment 2011) would likely appreciably reduce relevant POP exposures. However, we anticipate that long-term compliance practice is uncommon, given literature evidence of much shorter-term fish consumption guideline compliance among pregnant and nursing mothers (Teisl et al. 2011).

A further consideration for the effectiveness of POP-based fish advisories is that any minor chemical exposure benefit of short-term compliance is likely accompanied by a reduced intake of beneficial nutrients from fish, because many consumers following guidelines do in fact reduce overall fish intake rather than replace higher-contaminated fish with lesser-contaminated fish (Oken et al. 2012). Reductions in omega-3 polyunsaturated fatty acid (PUFA) intake, which is especially important for brain development of fetuses, newborns, toddlers, and children, are most critical (Kris-Etherton et al. 2002; Lee et al. 2009). Because up to 25% of omega-3 PUFAs are taken up with fish (Welch et al. 2006), markedly reducing fish consumption would also reduce their intake significantly.

Evidence that fish consumption advisory effectiveness rises and declines as contaminant emissions increase and decrease is of particular interest when considering the policy context in which food consumption guidelines for POPs are typically developed. Environmental contaminants identified as food-chain-bioaccumulation and biota-toxicity risks undergo rigorous study to understand any exposure concerns. Thus, issuing of advisories will often lag behind regulations to reduce emissions of the contaminant to the environment. Although it would be most advantageous to define consumption recommendations for chemicals undergoing increasing or peak emissions, the issuing of advisories well before undertaking emission reduction is unlikely.

Our model estimates suggest that the success of advisory adherence will be greatest for chemicals with a high metabolic transformation rate [e.g., polycyclic aromatic hydrocarbons (PAHs), and several PCDFs], because exposure reductions would be elevated if efficient contaminant removal occurred during the period of reduced chemical intake. This high impact of chemical half-life on guideline effectiveness is consistent with expectations, because a mother’s reduction in intake of chemicals with a 1-year transformation half-life for > 5 years should have a substantial effect on resultant exposures in her child. However, POP-based dietary advisories are often based on compounds characterized by high persistence, such as PCBs and PCDDs (Becker et al. 2007; Bhavsar et al. 2011). An important exception to this observation is methylmercury, which also possesses known bioaccumulating and toxic properties, and is the primary target of many published fish consumption guidelines (FDA 2004; Ontario Ministry of the Environment 2011). Methylmercury is eliminated from the human body with a half-life of 40–50 days (Stern 2005), and chemical space calculations for organic chemicals with human metabolic half-lives comparable to the human elimination half-life of methylmercury have suggested an appreciable potential impact of advisory compliance (see Supplemental Material, Figure S2). However, the modeling strategy we employed is unable to accurately reflect methylmercury bioaccumulation, given this contaminant’s unique interspecies conversion and protein-binding properties. Nevertheless, based on the human half-life of this compound, it is likely that short-term advisory compliance would have a greater impact on offspring exposure to methylmercury than on exposure to persistent organic chemicals.

The greater importance of advisories targeting methylmercury compared with those for POPs has also been previously addressed in the risk–benefit analysis literature (e.g., Cohen et al. 2005; Mozaffarian and Rimm 2006; Oken et al. 2012). In particular, Rheinberger and Hammitt (2012) recently published estimates of the potential prenatal health and development impacts of 2001 U.S. FDA fish advisories based on NHANES (National Health and Nutrition Examination Survey) estimates of the extent of maternal dietary fish substitution. Their model predicted “a modest benefit to newborns whose mothers shift consumption to comply with the current EPA/FDA advisory.” In accordance with our findings, they attributed nearly all of this benefit to reductions in methylmercury, whereas reductions in exposure to dioxin-like compounds were judged to have only marginal impact. Furthermore, these gains could be more than offset if overcompliance results in a lowering of omega-3 PUFA intake in non-target groups (Rheinberger and Hammitt 2012).

Notably, our calculations suggest that exposure to certain bioaccumulating compounds may increase following replacement of fish with other potentially contaminated foods. Particularly, substitution with animal products originating from the terrestrial food chain in Sweden or North America would likely result in greater exposure to more water-soluble persistent chemicals (2 < log *K_OW_* < 4) (Czub and McLachlan 2004a). Undeman et al. (2010) demonstrated that differences in both local environment and native diet had a substantial effect on the types of chemicals likely to bioaccumulate most in humans. This phenomenon was also supported by our model projections, because replacement of fish with beef resulted in predictions of greater contamination by persistent chemicals within two main areas of the chemical partitioning space approximately bordered by 2 < log *K*_ow_ < 6 and 7 < log *K*_oa_ < 9 and 8 < log *K*_ow_ < 10 and 7 < log *K*_oa_ < 9. Compounds with the former partitioning behavior include known persistent organic chemicals such as β-endosulfan, β-hexachlorocyclohexane, and hexachlorobenzene (Kelly and Gobas 2003). Given this, temporary adherence to current fish consumption advisories could prove even less effective in reducing exposure to persistent organics if replacement with different contaminated food maintained the intake level of contaminants or even increased the overall load. Inclusion of the best dietary substitutes for fish within advisories could mitigate this issue, by recommending low-risk food items that would prevent compensatory exposure to a different set of bioaccumulating compounds.

Finally, several limitations of our analysis based on inherent model assumptions merit mention. As discussed, our simulations rely on lipid partitioning to estimate food chain bioaccumulation, and we therefore cannot comment on the effectiveness of methylmercury-based fish advisory compliance, beyond predictions based solely on the elimination half-life of this chemical. Also, we did not address potential effects of predicted POP exposure reductions on health outcomes. Other limitations include the assumption that the entirety of contaminant load is derived from local food sources, that contaminated dietary proportions are unchanged throughout an individual’s lifetime, and that interindividual and intergenerational differences in reproductive behaviors or metabolic degradation rates are not considered. Collectively these assumptions likely limit the generalizability of absolute exposure reduction results to specific populations and contexts beyond the Baltic watershed, but our principal relative findings are expected to remain applicable to any temperate fish-eating community.

## Conclusion

We conducted a series of simulations to investigate the efficacy of maternal fish consumption advisory adherence in reducing exposures of offspring to PCB-153, as well as a range of hypothetical organic contaminants. Our results suggest that the main variables determining the impact on PCB-153 exposure are the degree of advisory compliance (to what extent fish is removed from the diet and the length of time for which the advisory is followed) and the time from peak contaminant emissions. Notably, PCB-153 exposure reductions were limited for the majority of dietary scenarios when maternal dietary adjustments lasted for either 1 year or 5 years before birth. This suggests that, for compounds with long half-lives, temporary fish advisory-related decreases in daily contaminant intake will not necessarily translate to appreciable decreases in maternal POP body burdens, a highly relevant parameter in determining prenatal dose. For the range of hypothetical chemicals assessed, the estimated effect of following guidelines was greatest and relatively uniform for chemicals within the region bounded by 2 < log *K*_ow_ < 11 and log *K*_oa_ > 7, a region including several highly bioaccumulative groups of compounds in humans (e.g., PCBs, PCDDs, polybrominated diphenyl ethers). Notably, the rate of chemical transformation in humans was a much greater influence on guideline effectiveness than the assumed degree of advisory compliance, because effectiveness increased as human transformation half-life decreased. Evaluation of fish consumption guidelines remains necessary for their improvement, and, as our study demonstrates, effective modeling practice may help inform this pursuit. Ultimately, for fish advisories targeting members of the general population sensitive to POP neurodevelopmental and reproductive deficits (i.e., expectant, pregnant, and nursing mothers), we recommend a focus on guidelines for chemicals with human elimination half-lives that are ≤ 1 year. We also propose that advisories include specific recommendations for healthy alternatives to fish, focusing on replacement food items that will reduce the likelihood that contaminant exposures will persist or even increase. Finally, a complete ban on fish consumption may be preferable to targeted, life stage–based fish consumption advisories when fish are heavily contaminated with substances that have long human elimination half-lives.

## Supplemental Material

(426 KB) PDF
