# Supplementary material for: Evaluating the Effectiveness of Fish Consumption Advisories: Modeling Prenatal, Postnatal, and Childhood Exposures to Persistent Organic Pollutants
Source: Environ Health Perspect. 2013 Dec 17;122(2):178–86. doi: 10.1289/ehp.1206380 (PMC3915257; doi:10.1289/ehp.1206380)
Supplement: (426 KB) PDF [file ehp.1206380.s001.pdf]

Environmental Health Perspectives doi:10.1289/ehp.1206380

Supplemental Material
Evaluating the Effectiveness of Fish Consumption Advisories: Modeling Prenatal, Postnatal, and Childhood Exposures to Persistent Organic Pollutants

Matthew J. Binnington, Cristina L. Quinn, Michael S. McLachlan, and Frank Wania

Table of Contents

Supplemental Material, Table S1. Estimated absolute and percent reductions in prenatal, postnatal, and childhood exposure to PCB-153 according to different 1-year maternal fish advisory compliance scenarios (relative to no change in fish consumption), and assuming time variant emissions................2

Supplemental Material, Table S2. Estimated absolute and percent reductions in prenatal, postnatal, and childhood exposure to PCB-153 according to different 5-year maternal fish advisory compliance scenarios (relative to no change in fish consumption), and assuming time variant emissions …...........5

Supplemental Material, Figure S1. Estimated absolute reductions in prenatal, postnatal, and childhood exposure to PCB-153 according to different maternal lifetime fish advisory compliance scenarios (relative to no change in fish consumption), and assuming steady state emissions................8

Supplemental Material, Figure S2. Estimated percent reductions in prenatal exposure to hypothetical chemicals with varying octanol-air (K_{OA}) and octanol-water partition (K_{OW}) coefficients at 25°C according to maternal total fish replacement with produce, and assuming a human biotransformation half-life (90 days) approaching that of methylmercury...9

Reference………………………………………………………………………….......................10

Supplemental Material, Table S1. Estimated absolute and percent reductions in prenatal, postnatal, and childhood exposure to PCB-153 according to different 1-year maternal fish advisory compliance scenarios (relative to no change in fish consumption), and assuming time variant emissions. Note the difference in concentration units between prenatal exposures (ng/g lipid) and postnatal and childhood exposures (ng/g lipid·y).

**Birth Year and 1-Year Fish Replacement Scenario**	**Prenatal Exposure Reduction ng/g lipid (%)**	**Postnatal Exposure Reduction ng/g lipid·y (%)**	**Childhood Exposure Reduction ng/g lipid·y (%)**
**1950**			
Default	4.4	8.5	12.5
Half-Beef	4.1 (6.4)	7.8 (7.8)	12.2 (2.6)
Half-Produce	4.0 (8.9)	7.7 (9.6)	12.1 (3.3)
All-Beef	3.7 (14.6)	7.1 (16.1)	11.8 (5.5)
All-Produce	3.6 (17.4)	6.8 (19.2)	11.7 (6.5)
**1955**			
Default	7.3	14.0	31.1
Half-Beef	7.0 (5.1)	13.2 (5.9)	30.6 (1.4)
Half-Produce	6.9 (5.9)	13.1 (6.7)	30.6 (1.6)
All-Beef	6.6 (10.1)	12.4 (11.4)	30.2 (2.6)
All-Produce	6.5 (11.7)	12.2 (13.1)	30.1 (3.1)
**1960**			
Default	16.7	32.4	71.7
Half-Beef	15.5 (7.2)	29.6 (8.5)	70.2 (2.1)
Half-Produce	15.3 (8.4)	29.2 (9.8)	70.0 (2.4)
All-Beef	14.2 (15.0)	26.9 (16.8)	68.9 (4.0)
All-Produce	13.8 (17.4)	26.0 (19.8)	68.4 (4.6)
**1965**			
Default	43.4	83.7	130.5
Half-Beef	40.2 (7.4)	76.7 (8.4)	126.7 (2.9)
Half-Produce	39.6 (8.8)	75.6 (9.7)	126.3 (3.2)
All-Beef	36.9 (15.0)	69.7 (16.7)	123.3 (5.5)
All-Produce	35.8 (17.5)	67.3 (19.6)	122.2 (6.4)

**Birth Year and 1-Year Fish Replacement Scenario**	**Prenatal Exposure Reduction ng/g lipid (%)**	**Postnatal Exposure Reduction ng/g lipid·y (%)**	**Childhood Exposure Reduction ng/g lipid·y (%)**
**1970**			
Default	86.4	165.4	227.8
Half-Beef	80.8 (6.5)	154.0 (6.9)	222.0 (2.6)
Half-Produce	80.0 (7.4)	151.6 (8.3)	220.8 (3.1)
All-Beef	75.3 (12.8)	141.6 (14.4)	215.8 (5.3)
All-Produce	73.6 (14.8)	138.0 (16.6)	213.9 (6.1)
**1975**			
Default	142.0	272.5	297.1
Half-Beef	132.0 (7.0)	251.8 (7.6)	286.3 (3.6)
Half-Produce	131.0 (7.7)	249.3 (8.5)	285.0 (4.1)
All-Beef	122.0 (14.1)	230.8 (15.3)	275.5 (7.3)
All-Produce	120.0 (15.5)	225.8 (17.2)	273.0 (8.1)
**1980**			
Default	226.0	428.7	300.6
Half-Beef	216.0 (4.4)	406.0 (5.3)	289.0 (3.9)
Half-Produce	214.0 (5.3)	402.9 (6.0)	287.7 (4.3)
All-Beef	205.0 (9.3)	383.3 (10.6)	278.0 (7.5)
All-Produce	202.0 (10.6)	377.3 (12.0)	275.0 (8.5)
**1985**			
Default	222.0	416.8	267.8
Half-Beef	215.0 (3.2)	402.8 (3.4)	260.6 (2.7)
Half-Produce	215.0 (3.2)	401.8 (3.6)	260.3 (2.8)
All-Beef	209.0 (5.9)	388.9 (6.7)	253.6 (5.3)
All-Produce	207.0 (6.8)	386.6 (7.3)	252.3 (5.8)
**1990**			
Default	189.0	355.5	224.1
Half-Beef	184.0 (2.6)	345.0 (3.0)	219.0 (2.3)
Half-Produce	184.0 (2.6)	343.6 (3.4)	218.3 (2.6)

**Birth Year and 1-Year Fish Replacement Scenario**	**Prenatal Exposure Reduction ng/g lipid (%)**	**Postnatal Exposure Reduction ng/g lipid·y (%)**	**Childhood Exposure Reduction ng/g lipid·y (%)**
All-Beef	179.0 (5.3)	333.8 (6.1)	213.2 (4.9)
All-Produce	178.0 (5.8)	331.8 (6.7)	212.4 (5.2)
**1995**			
Default	153.0	287.7	178.5
Half-Beef	149.0 (2.6)	278.9 (3.0)	174.2 (2.4)
Half-Produce	149.0 (2.6)	278.0 (3.4)	173.8 (2.6)
All-Beef	145.0 (5.2)	269.9 (6.2)	169.8 (4.9)
All-Produce	144.0 (5.9)	268.5 (6.7)	168.8 (5.5)
**2000**			
Default	119.0	222.9	133.6
Half-Beef	116.0 (2.5)	216.0 (3.1)	130.0 (2.7)
Half-Produce	115.0 (3.4)	215.5 (3.3)	130.0 (2.7)
All-Beef	113.0 (5.0)	209.3 (6.1)	126.9 (5.1)
All-Produce	112.0 (5.9)	208.3 (6.6)	126.3 (5.5)
**2005**			
Default	88.2	165.1	96.5
Half-Beef	86.1 (2.4)	160.4 (2.8)	94.2 (2.4)
Half-Produce	85.9 (2.6)	160.2 (3.0)	94.0 (2.6)
All-Beef	83.9 (4.9)	155.8 (5.6)	92.0 (4.7)
All-Produce	83.5 (5.3)	155.2 (6.0)	91.6 (5.1)
**2010**			
Default	63.5	118.8	69.3
Half-Beef	62.1 (2.2)	116.0 (2.4)	67.7 (2.3)
Half-Produce	61.9 (2.5)	115.6 (2.7)	67.6 (2.4)
All-Beef	60.6 (4.6)	112.7 (5.2)	66.2 (4.5)
All-Produce	60.3 (5.0)	112.0 (5.7)	65.9 (4.9)

Supplemental Material, Table S2. Estimated absolute and percent reductions in prenatal, postnatal, and childhood exposure to PCB-153 according to different 5-year maternal fish advisory compliance scenarios (relative to no change in fish consumption), and assuming time variant emissions. Note the difference in concentration units between prenatal exposures (ng/g lipid) and postnatal and childhood exposures (ng/g lipid·y). Also, the prenatal percent exposure reductions listed below correspond to those displayed in Figure 2.

**Birth Year and 5-Year Fish Replacement Scenario**	**Prenatal Exposure Reduction ng/g lipid (%)**	**Postnatal Exposure Reduction ng/g lipid·y (%)**	**Childhood Exposure Reduction ng/g lipid·y (%)**
**1950**			
Default	4.4	8.5	12.5
Half-Beef	3.4 (22.3)	6.6 (22.5)	11.5 (7.2)
Half-Produce	3.2 (26.0)	6.3 (26.2)	11.5 (8.3)
All-Beef	2.4 (44.4)	4.7 (44.9)	10.7 (14.4)
All-Produce	2.1 (51.6)	4.0 (52.4)	10.4 (16.8)
**1955**			
Default	7.3	14.0	31.1
Half-Beef	5.8 (21.1)	11.0 (21.5)	29.6 (4.5)
Half-Produce	5.5 (24.7)	10.5 (25.1)	29.4 (5.3)
All-Beef	4.2 (42.1)	8.0 (42.7)	28.2 (9.2)
All-Produce	3.7 (49.5)	7.0 (50.1)	27.7 (10.7)
**1960**			
Default	16.7	32.4	71.7
Half-Beef	12.7 (24.0)	24.5 (24.2)	68.0 (5.3)
Half-Produce	12.1 (27.5)	23.2 (28.3)	67.3 (6.2)
All-Beef	8.7 (47.8)	16.7 (48.5)	64.2 (10.5)
All-Produce	7.4 (55.6)	14.1 (56.5)	62.9 (12.3)
**1965**			
Default	43.4	83.7	130.5
Half-Beef	32.4 (25.3)	62.3 (25.5)	120.3 (7.9)
Half-Produce	30.6 (29.5)	58.8 (29.8)	118.6 (9.2)

**Birth Year and 5-Year Fish Replacement Scenario**	**Prenatal Exposure Reduction ng/g lipid (%)**	**Postnatal Exposure Reduction ng/g lipid·y (%)**	**Childhood Exposure Reduction ng/g lipid·y (%)**
All-Beef	21.5 (50.5)	40.9 (51.1)	110.0 (15.7)
All-Produce	17.8 (59.0)	33.8 (59.6)	106.8 (18.2)
**1970**			
Default	86.4	165.4	227.8
Half-Beef	66.0 (23.6)	125.9 (23.9)	209.0 (8.3)
Half-Produce	62.8 (27.3)	119.8 (27.6)	206.0 (9.6)
All-Beef	45.6 (47.2)	86.4 (47.8)	190.3 (16.5)
All-Produce	39.2 (54.6)	73.9 (55.3)	184.4 (19.1)
**1975**			
Default	142.0	272.5	297.1
Half-Beef	110.0 (22.5)	211.3 (22.4)	267.8 (9.9)
Half-Produce	106.0 (25.4)	203.1 (25.5)	263.7 (11.2)
All-Beef	79.1 (44.3)	149.8 (45.0)	238.4 (19.7)
All-Produce	70.7 (50.2)	133.6 (51.0)	230.7 (22.4)
**1980**			
Default	226.0	428.7	300.6
Half-Beef	176.0 (22.1)	332.8 (22.4)	255.6 (15.0)
Half-Produce	169.0 (25.2)	318.8 (25.6)	249.1 (17.1)
All-Beef	126.0 (44.2)	236.9 (44.7)	210.6 (30.0)
All-Produce	112.0 (50.4)	309.2 (51.2)	197.6 (34.3)
**1985**			
Default	222.0	416.8	267.8
Half-Beef	189.0 (14.9)	353.3 (15.2)	238.1 (11.1)
Half-Produce	185.0 (16.7)	347.3 (16.7)	235.1 (12.2)
All-Beef	155.0 (30.2)	289.9 (30.4)	208.3 (22.2)
All-Produce	149.0 (32.9)	277.5 (33.4)	202.5 (24.4)
**1990**			
Default	189.0	355.5	224.1

Birth Year and 5-Year Fish Replacement Scenario	Prenatal Exposure Reduction ng/g lipid (%)	Postnatal Exposure Reduction ng/g lipid·y (%)	Childhood Exposure Reduction ng/g lipid·y (%)
Half-Beef	165.0 (12.7)	309.4 (13.0)	202.8 (9.5)
Half-Produce	163.0 (13.8)	305.7 (14.0)	200.9 (10.4)
All-Beef	141.0 (25.4)	263.9 (25.8)	181.1 (19.2)
All-Produce	138.0 (27.0)	256.3 (27.9)	177.8 (20.7)
**1995**			
Default	153.0	287.7	178.5
Half-Beef	134.0 (12.4)	251.0 (12.7)	161.3 (9.6)
Half-Produce	133.0 (13.1)	247.7 (13.9)	159.9 (10.4)
All-Beef	115.0 (24.8)	214.3 (25.5)	144.2 (19.2)
All-Produce	112.0 (26.8)	208.3 (27.6)	141.3 (20.9)
**2000**			
Default	119.0	222.9	133.6
Half-Beef	104.0 (12.6)	194.3 (12.9)	120.3 (10.0)
Half-Produce	102.0 (14.3)	191.5 (14.1)	119.1 (10.9)
All-Beef	88.8 (25.4)	165.3 (25.8)	106.8 (20.1)
All-Produce	86.1 (27.6)	160.2 (28.1)	104.4 (21.9)
**2005**			
Default	88.2	165.1	96.5
Half-Beef	77.5 (12.1)	144.5 (12.5)	87.0 (9.9)
Half-Produce	76.6 (13.2)	142.6 (13.7)	86.2 (10.7)
All-Beef	66.9 (24.1)	124.4 (24.6)	77.5 (19.7)
All-Produce	65.0 (26.3)	120.7 (26.9)	75.8 (21.5)
**2010**			
Default	63.5	118.8	69.3
Half-Beef	56.4 (11.2)	105.2 (11.5)	62.8 (9.3)
Half-Produce	55.7 (12.3)	104.0 (12.5)	62.3 (10.1)
All-Beef	49.2 (22.5)	91.5 (23.0)	56.5 (18.5)
All-Produce	48.0 (5.0)	89.1 (25.0)	55.3 (20.1)

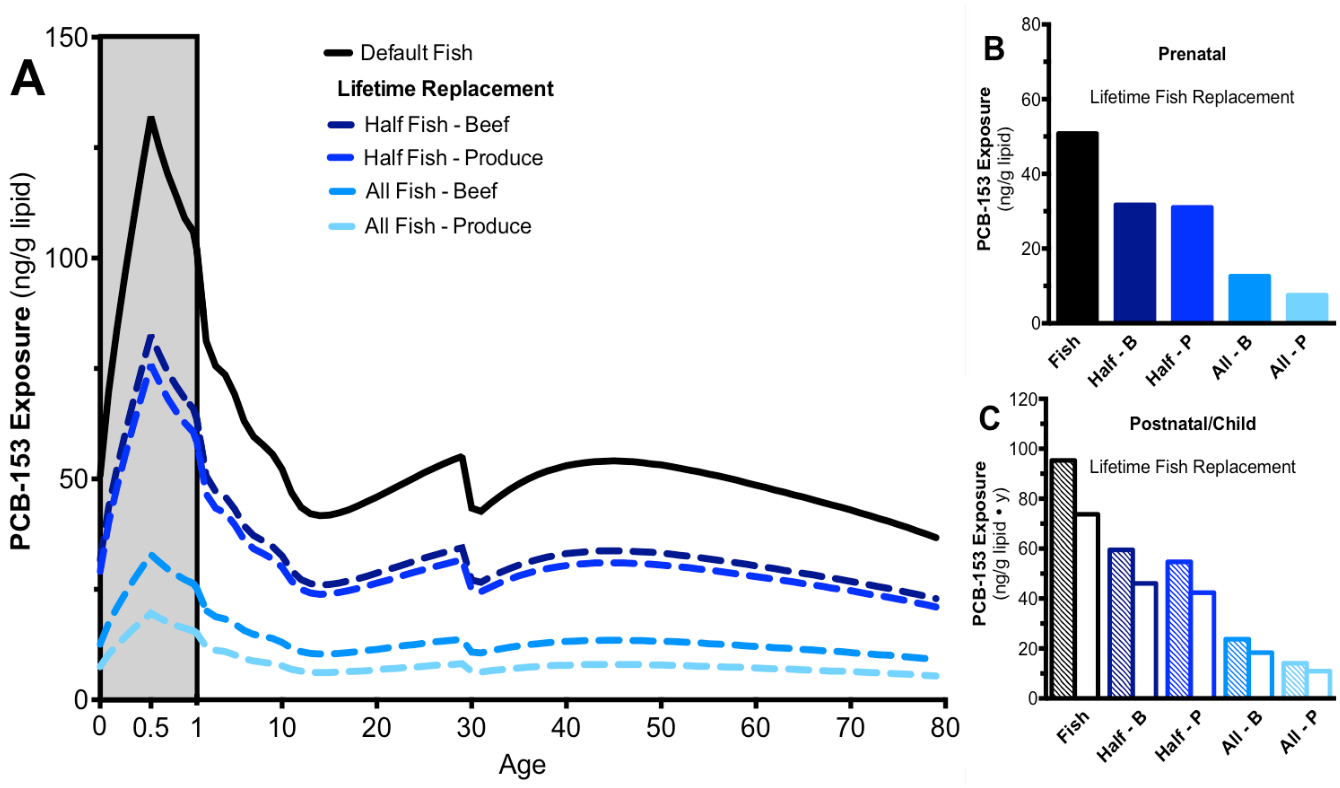

Supplemental Material, Figure S1. Estimated absolute reductions in prenatal, postnatal, and childhood exposure to PCB-153 according to different maternal lifetime fish advisory compliance scenarios (relative to no change in fish consumption), and assuming steady state emissions. Concentration age profiles for each lifetime compliance scenario are depicted in A). Note that exposure profiles are the same for all generations once the model reaches steady state, and thus the lifetime trends of PCB-153 contamination for a mother and her child under each scenario are depicted on the same graph. Prenatal peak exposures estimated by extent of compliance are depicted as solid bars in B). Postnatal and childhood exposures calculated on a per-year basis are displayed in C) as hatched and unfilled bars, respectively. Note that prenatal exposures in B) are point estimates of PCB-153 body burden at birth, while postnatal and childhood exposures in C) are the time-integrated areas under the curve during individuals' first 6 months and first 9 years of life, respectively.

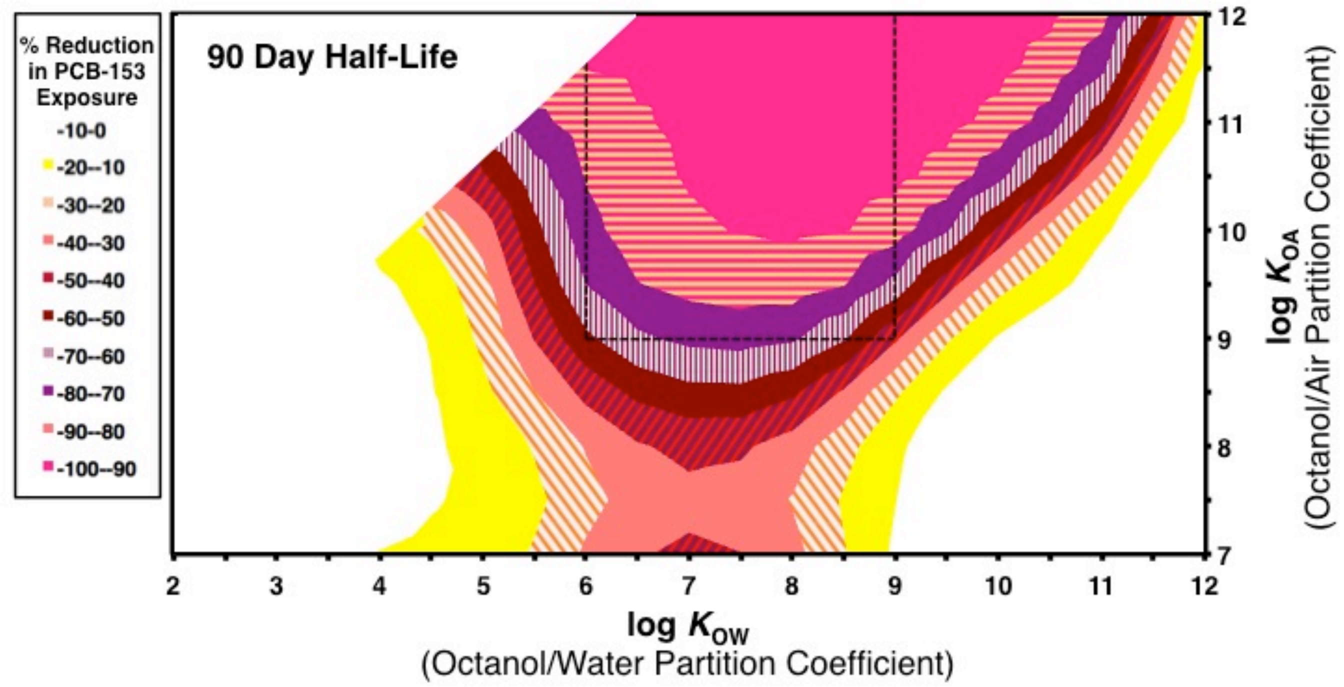

Supplemental Material, Figure S2. Estimated percent reductions in prenatal exposure to hypothetical chemicals with varying octanol-air (K_{OA}) and octanol-water partition (K_{OW}) coefficients at 25 °C according to maternal total fish replacement with produce, and assuming a human biotransformation half-life (90 days) approaching that of methylmercury. The graph represents the estimated percent reduction from the calculated default exposure following 5 years of replacing all fish intake with produce prior to childbirth. Note that when a reduction in chemical exposure is observed the percent change is assigned a negative value (-100 – 0%). Also, the region of enhanced fish to human bioaccumulation potential identified by Undeman et al. (2010) is denoted by dashed lines (log K_{OW} of 6 to 9 and log K_{OA} of 9 to 12).

Reference

Undeman E, Brown TN, Wania F, McLachlan MS. 2010. Susceptibility of human populations to environmental exposure to organic contaminants. Environ Sci Technol 44:6249-6255.
